# Diploid Genome Assembly of the Wine Grape Carménère

**DOI:** 10.1534/g3.119.400030

**Published:** 2019-03-28

**Authors:** Andrea Minio, Mélanie Massonnet, Rosa Figueroa-Balderas, Alvaro Castro, Dario Cantu

**Affiliations:** * Department of Viticulture and Enology, University of California Davis, Davis, CA 95618; † University of California-Davis Chile Life Sciences Innovation Center, Providencia, Chile

**Keywords:** genome assembly, heterozygosity, haplotype phasing, structural variation, *Vitis vinifera*

## Abstract

In this genome report, we describe the sequencing and annotation of the genome of the wine grape Carménère (clone 02, VCR-702). Long considered extinct, this old French wine grape variety is now cultivated mostly in Chile where it was imported in the 1850s just before the European phylloxera epidemic. Genomic DNA was sequenced using Single Molecule Real Time technology and assembled with FALCON-Unzip, a diploid-aware assembly pipeline. To optimize the contiguity and completeness of the assembly, we tested about a thousand combinations of assembly parameters, sequencing coverage, error correction and repeat masking methods. The final scaffolds provide a complete and phased representation of the diploid genome of this wine grape. Comparison of the two haplotypes revealed numerous heterozygous variants, including loss-of-function ones, some of which in genes associated with polyphenol biosynthesis. Comparisons with other publicly available grape genomes and transcriptomes showed the impact of structural variation on gene content differences between Carménère and other wine grape cultivars. Among the putative cultivar-specific genes, we identified genes potentially involved in aroma production and stress responses. The genome assembly of Carménère expands the representation of the genomic variability in grapes and will enable studies that aim to understand its distinctive organoleptic and agronomical features and assess its still elusive extant genetic variability. A genome browser for Carménère, its annotation, and an associated blast tool are available at http://cantulab.github.io/data.

Carménère (also known as Grand Vidure) is a historically and economically important wine grape (*Vitis vinifera* L.) cultivar with distinctive organoleptic and agronomical features (Huamán-Castilla *et al.* 2017). Carménère is an old French cultivar, which is thought to be derived from a cross between Cabernet Franc and Gros Cabernet (Boursiquot *et al.* 2009). It was widely planted in the Bordeaux regions of Graves and Médoc before the aphid-like soil-born pest *Phylloxera vastatrix* devastated French vineyards in the 19^th^ century. While almost extinct in France due to poor fruit set and late ripening, Carménère has well adapted to the Chilean climate and soil where it has become the flagship red wine grape with more than 10,000 hectares planted in most valleys throughout the country (Servicio Agrícola y Ganadero, https://www.sag.gob.cl/, 2016). Brought to Chile in the 1850s with other Bordeaux grapes just before phylloxera hit Europe, it was wrongly identified as Merlot until 1994 when the French ampelographer Jean Michel Boursiquot and, few years later, DNA fingerprinting (Hinrichsen *et al.* 2001) determined that the “Merlot Chileno” was instead Carménère (Pszczólkowski 2004; Richards 2006). A similar situation happened in Italy where it was confused with Cabernet Franc until 1991 (Caló *et al.* 1991). Carménère success is due to the peculiarity of its wines, which are deeply colored, with well-structured tannins, and distinctive aroma and flavor, that combine green, herbaceous features with fruity, spicy, berry-like notes (Casaubon *et al.* 2006; Fernández *et al.* 2007; Domínguez and Agosin 2010). Unlike most red wine cultivars, Carménère berries accumulates high concentration of methoxypyrazines, mainly 3-isobutyl-2-methoxypyrazine (IBMP) (Belancic and Agosin 2007), which confer the characteristic vegetal attributes in the resulting wines. The genetic bases of the phenological and compositional differences between Carménère and related varieties, such as Merlot, Cabernet Sauvignon, and Cabernet Franc, are not known.

Extensive structural variation between grape genotypes leads to significant unshared gene content between cultivars, which has been shown to contribute to varietal phenotypic characteristics (Venturini *et al.* 2013; Da Silva *et al.* 2013; Gambino *et al.* 2017; Zhou *et al.* 2018; Minio *et al.* 2019). Cultivar-specific genes have been discovered by whole-genome or transcriptome comparative analyses (Venturini *et al.* 2013; Da Silva *et al.* 2013; Gambino *et al.* 2017; Zhou *et al.* 2018; Minio *et al.* 2019). However, despite the relatively small genome size estimated at about 500 Mbp, the assembly of grape genomes is difficult because of the high level of heterozygosity (Minio *et al.* 2017). We recently reported that contiguous and accurate assemblies of grape genomes can be generated by assembling long Single-Molecule Real-Time (SMRT) sequencing reads using FALCON-Unzip, a diploid-aware assembler (Chin *et al.* 2016; Minio *et al.* 2017). In this work, we sequenced, assembled, and annotated the genome of Carménère clone 02 (VCR-702). As part of this project, we tested different combinations of assembly parameters, including variable sequencing coverage, to optimize the FALCON-Unzip pipeline and achieve the optimal contiguity and completeness of the assembly. Comparisons with other publicly available grape genomes identified structural variations and gene content differences in the Carménère genome.

## Methods & Materials

### Library preparation and sequencing

With permission from Vivai Cooperativi Rauscedo (Italy), we collected 1-2 cm-wide young leaves from Carménère clone 02 (equivalent to clone VCR 702) vines, maintained at Foundation Plant Services (FPS, University of California, Davis). High-molecular-weight genomic DNA (gDNA) was isolated using the method described in Chin *et al.* (2016). Genetic identity was confirmed with a standard set of microsatellite markers (Hinrichsen *et al.* 2001; This *et al.* 2004). DNA purity was evaluated with a Nanodrop 2000 spectrophotometer (Thermo Scientific, Hanover Park, IL, USA), DNA quantity with the DNA High Sensitivity kit on a Qubit 2.0 Fluorometer (Life Technologies, Carlsbad, CA, USA), and DNA integrity by pulsed-field gel electrophoresis. gDNA was cleaned with 0.45x AMPure PB beads (Pacific Biosciences, Menlo Park, CA, USA) before library preparation. SMRTbell template was prepared with 15 µg of sheared DNA using SMRTbell Template Prep Kit (Pacific Biosciences, Menlo Park, CA, USA) following the manufacturer’s instructions. For size selection, 30 µl of SMRTbell template were loaded on a Sage Blue Pippin (Sage Science, Beverly, MA, USA) and size-selected with a cutoff range of 17-50 Kbp. The size-selected library was cleaned with 1x AMPure PB beads. After DNA damage repair, the library were cleaned again with 1x AMPure PB beads. A total of 62 SMRT cells were sequenced on a PacBio RS II using P6/C4 chemistry, generating 6,615,332 reads for a total of ∼56 Gbp. DNA-seq libraries were prepared using the Kapa LTP library prep kit (Kapa Biosystems, Wilmington, MA, USA) and evaluated for quantity and quality with the High Sensitivity chip on a Bioanalyzer 2100 (Agilent Technologies, Santa Clara, CA, USA). A total of 100,190,577 DNA fragments were sequenced into 2x150bp reads on an Illumina HiSeq4000 (DNA Technology Core Facility, University of California, Davis).

### Genome assembly

Assembly of SMRT reads was performed with a customized FALCON-Unzip pipeline (v.2017.06.28-18.01; Chin *et al.* 2016), whose codes can be found at https://github.com/andreaminio/FalconUnzip-DClab. Prior to error correction, repeats were marked using the TANmask and REPmask modules from the DAmasker (Myers 2014). Repeats were marked also on error-corrected reads before assembly with FALCON. This additional repeat masking step increased assembly contiguity by 20% and decreased the computational time required for assembly by about 6%. To test the impact of sequencing coverage on FALCON assembly, raw SMRT reads were down-sampled randomly using seqtk (v.1.2-r101-dirty; https://github.com/lh3/seqtk) at theoretical 100x, 75x, 50x, 25x, 10x and 5x coverages. All datasets were assembled with FALCON without masking of corrected reads for all coverage combinations and with masking for full dataset down to 50x of coverage. As sequencing coverage influences error correction, we repeated the assembly on datasets created by down-sampling the error-corrected reads at 25x, 20x, 15x, 10x, and 5x coverages. Hybrid error correction was also tested to improve sequence accuracy of low-coverage dataset with short reads. Hybrid error correction was performed using LoRDEC (v.0.7 with GATB v.1.2.2; Salmela and Rivals 2014) with 1, 5 or 9 iterations over 50x, 25x, 10x and 5x datasets. FALCON-Unzip was performed on all 82 datasets with multiple assembly parameters (*i.e.*, read length retention threshold, self-alignment diagonal bands of width, read correlation rate, k-mer size and number of hits) for a total of 1,027 independent assemblies that were evaluated for contiguity and completeness (Table S1). Gene space completeness in the assembly was assessed by alignment of the complete PN40024 V1 genes (http://genomes.cribi.unipd.it/DATA/) on primary contigs using GMAP (v.2015-09-29; Wu and Watanabe 2005) with parameters “-B 4 -x 30 -f2” and discarding mappings with translocations. Haplotype phasing was carried out with Unzip and default parameters (Chin *et al.* 2013). Primary contigs and haplotigs were polished with Arrow (from ConsensusCore2 v.3.0.0). Primary contigs were scaffolded using SSPACE Longreads (v.1.1; Boetzer and Pirovano 2014), followed by gap closing with PBJelly (PBsuite v.15.8.4; English *et al.* 2012, 2014). Gene space completeness of the final assembly was assessed with BUSCO (v.3; Simão *et al.* 2015).

### Genome annotation

Repetitive sequences were identified with RepeatMasker (v.open-4.0.6; Smit *et al.* 2013) using a custom *V. vinifera* repeat library described in Minio *et al.* (2019). Repeats were masked prior to gene prediction. Protein-coding genes were predicted with EVM (v.1.1.1; Haas *et al.* 2008) using as input: (i) *ab initio* predictions from SNAP (v.2006-07-28; Korf 2004), Augustus (v.3.0.3; Stanke *et al.* 2006), GeneMark-ES (v.4.32; Lomsadze *et al.* 2005), GlimmerHMM (v.3.0.4; Majoros *et al.* 2004), and GeneID (v.1.4.4; Parra *et al.* 2000) trained on Cabernet Sauvignon; (ii) *ab initio* predictions of Augustus trained on BUSCO dataset; (iii) as experimental evidence, proteins from Swissprot viridiplantae (downloaded on 2016.03.15), mapped with Exonerate (v.2.2.0; Slater and Birney 2005); (iv) as transcriptional evidence, *Vitis* ESTs and flcDNAs (downloaded on 2016.03.15), *Vitis vinifera* PN40024 V1 CDS (http://genomes.cribi.unipd.it/DATA/), Tannat (TSA GAKH01.1) and Corvina (TSA PRJNA169607) transcriptomes, and Cabernet Sauvignon corrected Iso-Seq reads (SRP132320); (v) PASA (v.2.1.0; Haas *et al.* 2003, 2008; Campbell *et al.* 2006) predicted gene models based on the transcriptional evidences from (iv). Functional annotations were assigned based on homology with proteins in the RefSeq plant protein database (downloaded on 2017.01.17) and on functional domains identified with InterProScan (v.5; Jones *et al.* 2014; Table S2). Gene content variability between cultivars was assessed by alignment of the Carménère genes onto the PN40024 genome 12X.v2 (Canaguier *et al.* 2017) and Cabernet Sauvignon clone 08 genome (Chin *et al.* 2016) using GMAP (v.2015-09-29; Wu and Watanabe 2005) with identity and coverage >80%. Homology with Corvina, Tannat, and Nebbiolo transcripts was determined by blastn search of hits with reciprocal identity and coverage greater than 80%. Structural comparisons between assemblies were performed with MUMMER (v.4.0; Marçais *et al.* 2018) and variant impacts were annotated with SnpEff (v.4.3m; Cingolani *et al.* 2012).

The phylogenetic tree illustrating the relation between the different *VviOMT* alleles was obtained using the Neighbor-Joining method (Saitou and Nei 1987). The percentage of replicate trees in which the associated sequences clustered together in the bootstrap test (1,000 replicates) are shown next to the branches. The analysis involved 330 amino acid positions. All positions containing gaps and missing data were eliminated. Evolutionary analyses were conducted in MEGA7 (Kumar *et al.* 2016).

### Data availability

Raw sequences are available at NCBI (Bioproject PRJNA517468). Other relevant data, such as genome sequence, gene and protein sequences, gene and repeat coordinates and annotation, along with a genome browser and a blast tool, are available at http://cantulab.github.io/data.html. Supplemental material available at Figshare: https://doi.org/10.25387/g3.7666886.

## Results and Discussion

### Assembly of the Carménère genome

**Figure 1:**
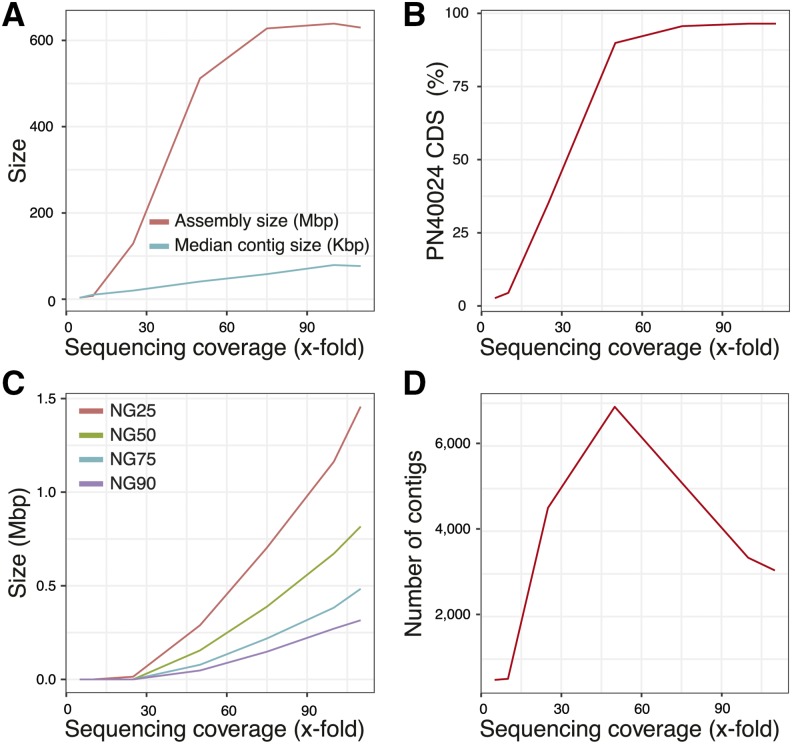
Impact of sequencing coverage on (A) assembly size, (B) completeness of the gene space, (C) assembly contiguity, and (D) assembly fragmentation. Best assemblies at each coverage were plotted.

The genome of *Vitis vinifera* cv. Carménère clone 02 was sequenced at 115x coverage using Single Molecule Real Time (SMRT; Pacific Biosciences) technology. The long reads (N50 = 13.1 Kbp) were assembled into primary contigs and haplotigs using the diploid-aware assembler FALCON-Unzip (Chin *et al.* 2016). As detailed in the Methods section, to optimize assembly we systematically tested the effect of sequencing coverage, type of error correction, and assembly parameters on genome contiguity and completeness (Figure 1; Table S1). We also tested the addition of an extra step of repeat masking of the error-corrected reads prior to the assembly step. The most contiguous assembly was obtained with full SMRT reads dataset, non-hybrid error correction, masking of repeats in corrected reads, and a minimum corrected reads retention threshold of 7.5 Kbp (Table 1). While optimal assembly and gene space completeness were achieved already at coverage 75x and 50x, respectively (Figure 1A&B), assembly contiguity increased exponentially with increasing coverage (Figure 1C&D). These results suggest that even more contiguous assemblies could have been produced at sequencing coverage greater than 115x likely because of the larger number of long reads used in the assembly. These results also show that under 25x of coverage, assembly contiguity and gene space completeness are compromised even when reads are error-corrected with over 100x coverage of long reads or a hybrid error correction using short reads is applied. The most contiguous and complete primary assembly was scaffolded into 1,411 scaffolds covering 622.8 Mbp (N50 = 1.04 Mbp) with a maximum length of 5.9 Mbp and with as few as 857 Kbp in gaps (0.14%). As expected, haplotigs were more fragmented and covered only 420.3 Mbp (7,969 contigs, N50 = 89.6 Kbp) (Table 1). The assembly contained 93.3% of the complete universal single-copy orthologs (BUSCO) genes. As observed previously in Cabernet Sauvignon (Chin *et al.* 2016) and Chardonnay (Zhou *et al.* 2018), the size of the assembly constructed with FALCON-Unzip is larger than the expected genome size (∼500 Mbp) likely due to the retention of both copies of some heterozygous regions in the primary contigs. Nonetheless, the total assembly size (primary + haplotigs of 1.04 Gbp) was twice the expected haploid genome size, which suggests that sequences of all homologous chromosomes are represented in the final assembly. This was confirmed by the presence in the total assembly of an average of 2.07 ± 0.86 copies for each of the PN40024 genes.

**Table 1:** Summary statistics of the Carménère genome assembly

	Primary assembly	Haplotigs
**Assembly length**	622,795,289 bp	420,345,460 bp
**Number of sequences**	1,411	7,969
**Average length**	441,386 bp	52,748 bp
**Maximum length**	5,905,621 bp	743,383 bp
**N50 length (index)**	1,039,379 bp (168)	89,565 bp (1029)
**Total gap length**	0.14%	0.00%
**Repetitive content**	308.9 Mbp (49.6%)	192 Mbp (45.7%)
**Number of genes**	40,684	32,425

### Annotation of the Carménère genome

**Figure 2:**
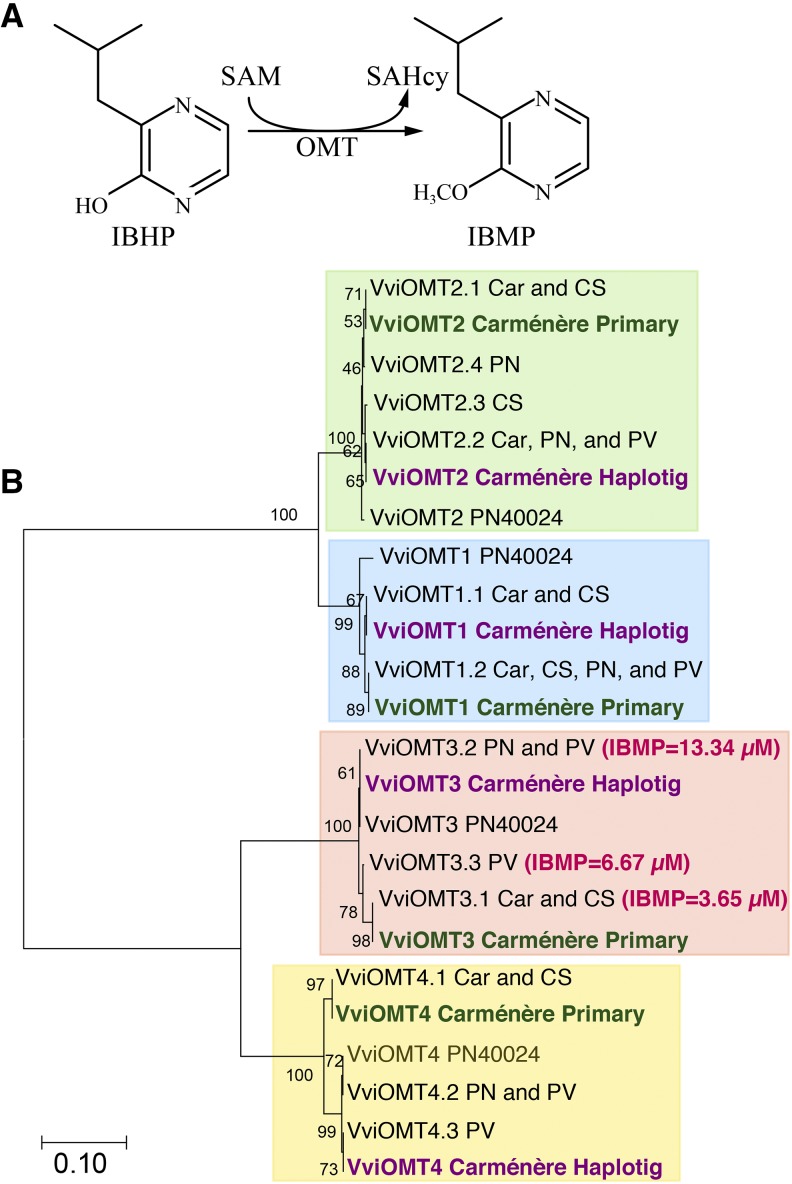
Phylogenetic tree of *O*-methyltransferases (OMTs). (A) Graphical representation of the last and critical step of the methoxypyrazine biosynthesis pathway, where a S-adenosyl-l-Met (SAM)-dependent O-methyltransferase (OMT) converts 3-isobutyl-2-hydroxypyrazine (IBHP) into 3-isobutyl-2-methoxypyrazine (IBMP) producing S-Adenosyl-l-homo-Cys (SAHcy). (B) Phylogenetic tree illustrating the relation between the different alleles of the VviOMT clade. The VviOMT clade was divided into 4 subclades as reported in Guillaumie *et al.* (2013) (VviOMT1, VviOMT2, VviOMT3 and VviOMT4). The percentage of replicate trees in the bootstrap test clustering the associated sequences are shown next to the branches. Sequences of the different alleles identified in Carménère genome annotation are indicated in bold, green for the allele reported in the primary sequences and violet for the one in the haplotigs. These sequences were compared to PN40024 annotation and the VviOMT alleles reported for different genotypes in Guillaumie *et al.* (2013). The acronyms reported indicate the original genotype: Carménère (Car), Cabernet Sauvignon (CS), Pinot Noir (PN), Petit Verdot (PV); where more than one genotype was sharing the same allele, a coma separated list is reported. The amount of IBMP produced *in vitro* by the three recombinant VviOMT3 proteins is indicated in parenthesis (Supplementary data from Guillaumie *et al.* 2013).

Forty eight percent of the assembled sequences in the primary scaffolds and haplotigs were classified as repetitive, mostly due to LTR transposable elements of the Gypsy (23.6% of repetitive content) and Copia (8.4% of repetitive content) families. A total of 73,109 protein-coding genes were found in the assembly, 40,684 in the primary assembly, and 32,425 in the haplotigs. The predicted transcriptome represented 95% of the BUSCO genes. All genes had at least one homolog plant protein in the RefSeq database, 69,918 (95.6%) had an InterPro match, 53,556 (73.3%) were assigned a gene ontology (GO) term, and 8,449 were associated with an enzyme code (EC; Table S2). One of the key aromatic compounds in Carménère are methoxypyrazines (MP), which impart the characteristic herbaceous, green, vegetal sensory attributes to Carménère wines. With wide variability among clones, Carménère grapes can accumulate high IBMP concentrations (5.0 to 44.4 ng/L; Belancic and Agosin 2007). The last step of the MP biosynthesis pathway consists in the conversion of 3-isobutyl-2-hydroxypyrazine (IBHP) into IBMP by a S-adenosyl-l-Met (SAM)-dependent *O*-methyltransferase (OMT; Figure 2A). Four *VviOMT* genes have been found in the grape genome, among which *VviOMT3* is considered the major determinant of IBMP production during berry ripening (Dunlevy *et al.* 2013; Guillaumie *et al.* 2013). We could identify in the Carménère genome all four members of the *VviOMT* gene family. For each *VviOMT*, both alleles were represented in the Carménère assembly, one in the primary contigs and one in the haplotigs, confirming the completeness of the diploid assembly (Figure 2B; Table S3). Interestingly, the two alleles of *VviOMT3* were polymorphic, with one allele closer to Cabernet Sauvignon (*VviOMT3.1)*, likely derived from Cabernet Franc, and one closer to the allele found in Pinot Noir and Petit Verdot (*VviOMT3.2*). The latter allele was shown to be a strong IBMP producer *in vitro*, which may explain the greater accumulation of IBMP in Carménère than in Cabernet Sauvignon (Belancic and Agosin 2007). Interestingly, *VviOMT3.2* was not found in a Carménère clone cultivated in Bordeaux (Guillaumie *et al.* 2013), which may explain the variability in IBMP accumulation among Carménère clones (Belancic and Agosin 2007). Because *OMT* expression was shown to play a critical role in IBMP accumulation (Guillaumie *et al.* 2013), further genetic analysis of the two alleles across multiple Carménère clones should be combined with gene expression measurements during ripening in order to determine the role of the different alleles in the accumulation of IBMP in Carménère grapes.

### Sequence and structural heterozygosity in the Carménère genome

**Figure 3:**
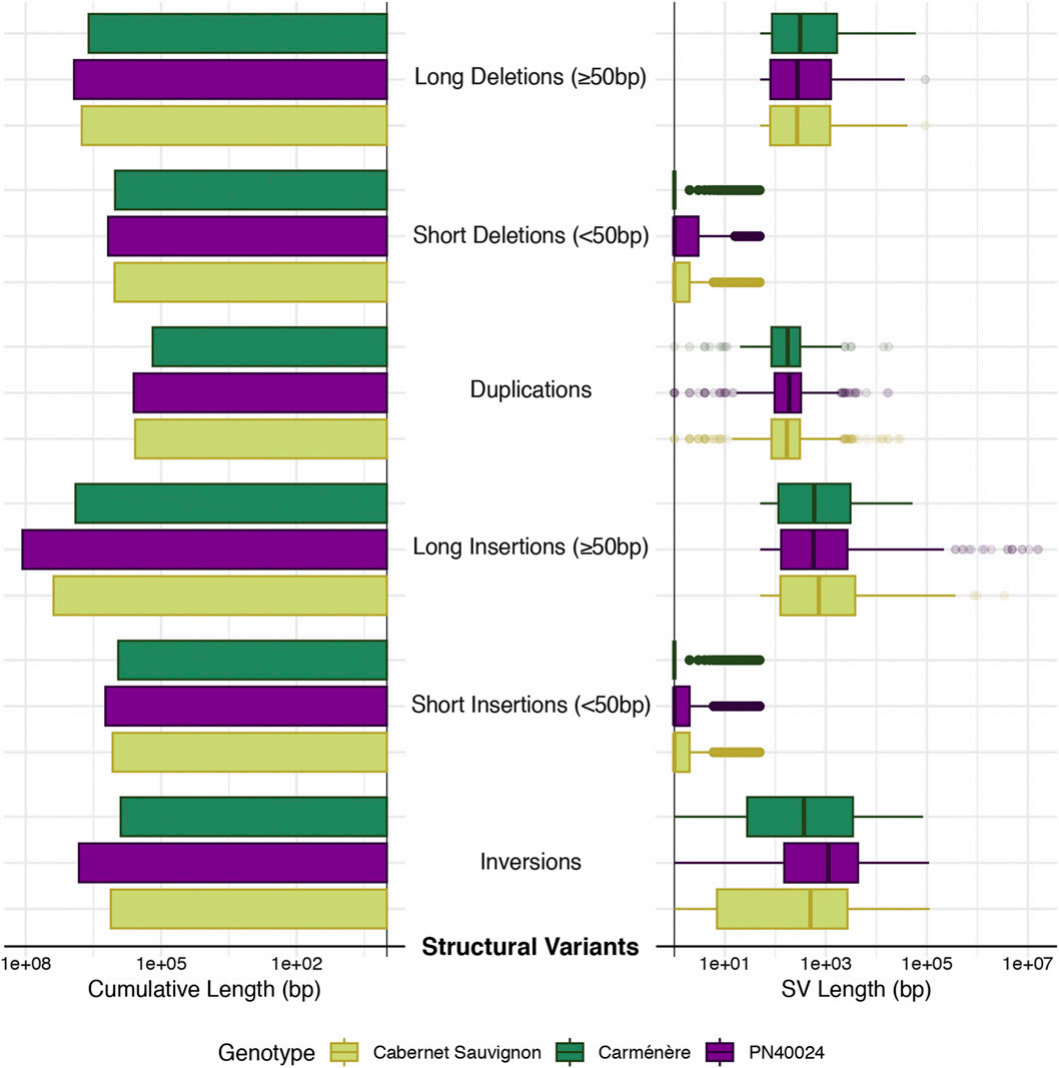
Size and length distribution of structural variants identified in the Carménère primary scaffolds when compared to Carménère haplotigs, PN40024 (chromosomes), and Cabernet Sauvignon (primary assembly).

Comparisons between the primary assembly and haplotigs revealed 1,506,269 SNPs (2.41 SNPs/Kbp), 1,127,746 INDELs (<50bp), and 6,159 deletion/insertion events (≥50bp), representing a total variation of 15.3 Mbp between the two haplotypes (2.5% and 3.6% of the primary assembly and haplotigs, respectively; Table 2; Figure 3). A total of 50,619 SNPs were identified in coding sequences (28,032 non-synonymous and 22,587 synonymous), resulting in the introduction of premature stop codons in 421 genes. Among the affected genes were four genes involved in the phenylpropanoid/flavonoid biosynthetic pathway: a 4-coumarate-CoA ligase, a chalcone synthase, a flavonoid 3′,5′-hydroxylase, and a flavonol synthase (Castellarin *et al.* 2012). The large deletions/inversions (≥50bp) involved 63 complete genes. A larger number of potentially hemizygous genes (2,844 sequences; identity and coverage >80%) was found by alignment of the haplotigs’ genes onto the primary assembly.

**Table 2:** Structural variants identified in the Carménère primary scaffolds when compared to Carménère haplotigs, PN40024 (chromosomes), and Cabernet Sauvignon (primary assembly)

	Carménère Primary Scaffolds					
	Carménère Haplotigs		PN40024		Cabernet Sauvignon	
	*Count*	*Total length*	*Count*	*Total length*	*Count*	*Total length*
**SNPs**	1,506,269	1,506,269	3,917,352	3,917,352	2,449,007	2,449,007
**Short Insertions (<50bp)**	503,729	891,412	617,760	1,712,482	499,827	1,185,538
**Short Deletions (<50bp)**	624,017	1,046,233	452,437	1,489,269	408,465	1,073,441
**Long Insertions (≥50bp)**	3,412	7,875,979	11,436	117,627,739	6,394	24,115,347
**Long Deletions (≥50bp)**	2,747	4,021,742	6,986	8,466,055	4,599	5,708,870
**Duplication Contraction**	186	72,918	736	256,328	396	134,216
**Duplication Expansion**	229	80,725	541	148,123	405	241,869
**Inversions**	170	784,551	1,551	6,594,750	460	1,300,866

### Genome structure and gene content comparison with other publicly available grapevine genomes and transcriptomes

The Carménère assembly was compared with the genome sequences of PN40024 (Jaillon *et al.* 2007; Canaguier *et al.* 2017) and Cabernet Sauvignon (Chin *et al.* 2016) to assess the extent and nature of the genetic diversity between these publicly available genomes. Direct comparison of genomic sequences identified many more variants between Carménère and PN40024 (3,917,352 SNPs; 1,070,197 <50bp INDELs) than between Carménère and Cabernet Sauvignon (2,449,007 SNPs; 908,292 <50bp INDELs) (Table 2; Figure 3). This result likely reflects the fact that Carménère and Cabernet Sauvignon share one of their parents (Bowers and Meredith 1997; Boursiquot *et al.* 2009). As expected, variants were detected at greater frequency in the intergenic space and introns than in exons in both comparisons (Table 2). SNPs and small INDELs were predicted to have deleterious impact on 8,988 and 7,835 Carménère genes when compared to PN40024 and Cabernet Sauvignon, respectively. We also identified large structural variants (SVs) between the three cultivars. A larger number of SVs was identified between Carménère and PN40024 (21,250 SVs) involving 133.1 Mbp (21.4%) of the Carménère primary assembly. Relative to the Cabernet Sauvignon genome, we could identify 12,254 SVs involving 31.5 Mbp of the Carménère assembly. Some of the large SVs intersected the gene space, which resulted in the absence of 494 and 253 Carménère genes in the PN40024 and Cabernet Sauvignon genomes, respectively. These SVs, and potentially additional undetected ones, may have contributed to the differences in gene content between Carménère and the other cultivars. About 2% of the Carménère genes (1,561) were not found in PN40024 and 0.61% (449) were not found in the Cabernet Sauvignon genome. A total of 198 genes were not found in any other available *V. vinifera* transcriptomes (Venturini *et al.* 2013; Da Silva *et al.* 2013; Gambino *et al.* 2017). These putative cultivar-specific genes comprised three sesquiterpene synthases, including two (-)-germacrene D synthases, which may be involved in terpenoid biosynthesis and grape aroma (Lücker *et al.* 2004). Carménère-specific genes also included: a sugar transporter ERD6-*like* 6 gene (early-responsive to dehydration) and an inositol-3-phosphate synthase-encoding gene, both potentially associated with water-deficit stress response (Büttner 2007; Yamada *et al.* 2010; Conde *et al.* 2015); seven Nucleotide Binding Site/Leucine-Rich Repeat (NBS-LRR) genes, five Serine/Threonine kinase genes, three LRR receptor-like kinase genes, that belong to three classes of resistance genes (Di Gaspero and Cipriani 2003; Kruijt *et al.* 2005). This level of unshared gene content is similar to what has been reported in previous works that compared other cultivars with PN40024 (Da Silva *et al.* 2013; Minio *et al.* 2019). Further work is necessary to determine whether some of these “private” Carménère genes contribute to its distinctive organoleptic and agronomical features. Further genetic analyses of Carménère will benefit from the availability of this high-quality genome assembly.
